# Plectin isoform P1b and P1d deficiencies differentially affect mitochondrial morphology and function in skeletal muscle

**DOI:** 10.1093/hmg/ddv184

**Published:** 2015-05-27

**Authors:** Lilli Winter, Andrey V. Kuznetsov, Michael Grimm, Anikó Zeöld, Irmgard Fischer, Gerhard Wiche

**Affiliations:** 1 Max F. Perutz Laboratories, Department of Biochemistry and Cell Biology, University of Vienna, 1030 Vienna, Austria,; 2 Institute of Neuropathology, University Hospital Erlangen, 91054 Erlangen, Germany and; 3 Cardiac Surgery Research Laboratory, Department of Cardiac Surgery, Innsbruck Medical University, 6020 Innsbruck, Austria

## Abstract

Plectin, a versatile 500-kDa cytolinker protein, is essential for muscle fiber integrity and function. The most common disease caused by mutations in the human plectin gene, epidermolysis bullosa simplex with muscular dystrophy (EBS-MD), is characterized by severe skin blistering and progressive muscular dystrophy. Besides displaying pathological desmin-positive protein aggregates and degenerative changes in the myofibrillar apparatus, skeletal muscle specimens of EBS-MD patients and plectin-deficient mice are characterized by massive mitochondrial alterations. In this study, we demonstrate that structural and functional alterations of mitochondria are a primary aftermath of plectin deficiency in muscle, contributing to myofiber degeneration. We found that in skeletal muscle of conditional plectin knockout mice (MCK-Cre/cKO), mitochondrial content was reduced, and mitochondria were aggregated in sarcoplasmic and subsarcolemmal regions and were no longer associated with Z-disks. Additionally, decreased mitochondrial citrate synthase activity, respiratory function and altered adenosine diphosphate kinetics were characteristic of plectin-deficient muscles. To analyze a mechanistic link between plectin deficiency and mitochondrial alterations, we comparatively assessed mitochondrial morphology and function in whole muscle and teased muscle fibers of wild-type, MCK-Cre/cKO and plectin isoform-specific knockout mice that were lacking just one isoform (either P1b or P1d) while expressing all others. Monitoring morphological alterations of mitochondria, an isoform P1b-specific phenotype affecting the mitochondrial fusion–fission machinery and manifesting with upregulated mitochondrial fusion-associated protein mitofusin-2 could be identified. Our results show that the depletion of distinct plectin isoforms affects mitochondrial network organization and function in different ways.

## Introduction

Mitochondria perform a multitude of cellular activities that are essential for the life and death of cells, such as energy production in the form of ATP, cell respiration, fatty acid and amino acid metabolism and the regulation of various ions, in particular calcium. Also, mitochondria are central in apoptosis, production of reactive oxygen species associated with oxidative stress and cellular signaling. Importantly, the cellular arrangement, morphology, regulation of function and several other activities of mitochondria strongly depend on their interactions with elements of the cytoskeleton, albeit the molecular mechanisms involved are hardly understood (1,2).

One interesting candidate for mediating interactions between the cytoskeleton and mitochondria is the cytolinker protein plectin, which belongs to a group of structurally related proteins, referred to as the plakin protein family (3,4). Plectin is a highly versatile protein acting as a mechanical linker between the intermediate filament (IF) network and various cytoskeletal structures and organelles, including the subplasma membrane skeleton, specialized junctional complexes, such as focal adhesions, desmosomes, hemidesmosomes, the neuromuscular junctions and junctional complexes of Schwann cells, Z-disks and the nuclear lamina. Moreover, it mediates the crosstalk of IFs with the actin and microtubule cytoskeleton (5). Plectin's versatility is in part due to complex splicing events in the N-terminal region of its gene giving rise to 11 alternatively spliced isoforms containing different first exons (1–1j) (5,6). Some of these isoforms show a tissue-specific distribution (6,7), and distinct subcellular targeting has been demonstrated by forced expression of full-length and truncated plectin versions (8,9). Previous studies suggested that, in skeletal muscle, the four major plectin isoforms expressed are crucial for the integrity of myofibers by specifically targeting and anchoring desmin IF networks to Z-disks (plectin isoform 1d, P1d), costameres (P1f), mitochondria (P1b) and the nuclear/sarcoplasmic reticulum (SR) membrane system (P1). On a single cell level, plectin deficiency has been reported to lead to shape changes of mitochondria, manifesting as an elongation of mitochondrial networks in plectin-deficient fibroblasts (10) and myoblasts (11).

The most common disease caused by mutations in the human plectin gene (*PLEC*, NM_000445), epidermolysis bullosa simplex with muscular dystrophy (EBS-MD; OMIM 226670), is characterized by severe skin blistering and progressive muscular dystrophy. In skeletal muscle samples from EBS-MD patients (12,13), as well as from muscle-restricted conditional plectin knockout mice (MCK-Cre/cKO) (14), remarkable mitochondrial alterations have been observed, including altered cristae structure and massive subsarcolemmal aggregation of mitochondria, and the presence of fibers with attenuated levels of mitochondria (15). For instance, the analysis of a patient carrying a 16-bp insertion mutation close to the IF-binding site of plectin revealed a severely disorganized myogenic IF cytoskeleton, associated with abnormal positioning of mitochondria, subcellular regions exhibiting diminished organelles and a reduced activity of respiratory chain Complexes I and II (12). Moreover, the ultrastructural analysis of skeletal muscle specimens derived from patients where EBS-MD was found to be associated with myasthenic syndrome (EBS-MD-MyS) unraveled clustering of mitochondria under the sarcolemma, near nuclei or in more central parts of the myofibers, leaving large parts of adjacent fiber regions depleted of mitochondria (16). Similarly, an accumulation of thread-like mitochondria at neuromuscular junctions was reported for another patient carrying a homozygous plectin mutation combined with a mutation in the gene *CHRNE*, which encodes a subunit of the acetylcholine receptor (13).

Presently, it is not known whether the mitochondrial pathology observed in plectinopathy patients and MCK-Cre/cKO mice represents secondary effects attributable to myofiber degeneration and muscle dystrophy or reflects a more specific mechanism involving a particular isoform of plectin. To better understand the mechanism(s) behind mitochondrial defects in plectinopathies and to dissect the role of individual isoforms of plectin, we comparatively analyzed morphological and functional characteristics of mitochondrial networks in muscle tissue and teased muscle fibers from wild-type, striated muscle-restricted (MCK-Cre/cKO) and two isoform-specific (P1b and P1d) knockout mouse lines. Our analysis revealed that the uncoupling of IFs from mitochondria through specific ablation of plectin isoform P1b causes severe mitochondrial dysfunctions including the upregulation of mitochondrial fusion-associated protein mitofusin-2 (Mfn-2).

## Results

### Mitochondrial alterations in plectin-deficient skeletal muscle occur from early age on

**Figure 1.**
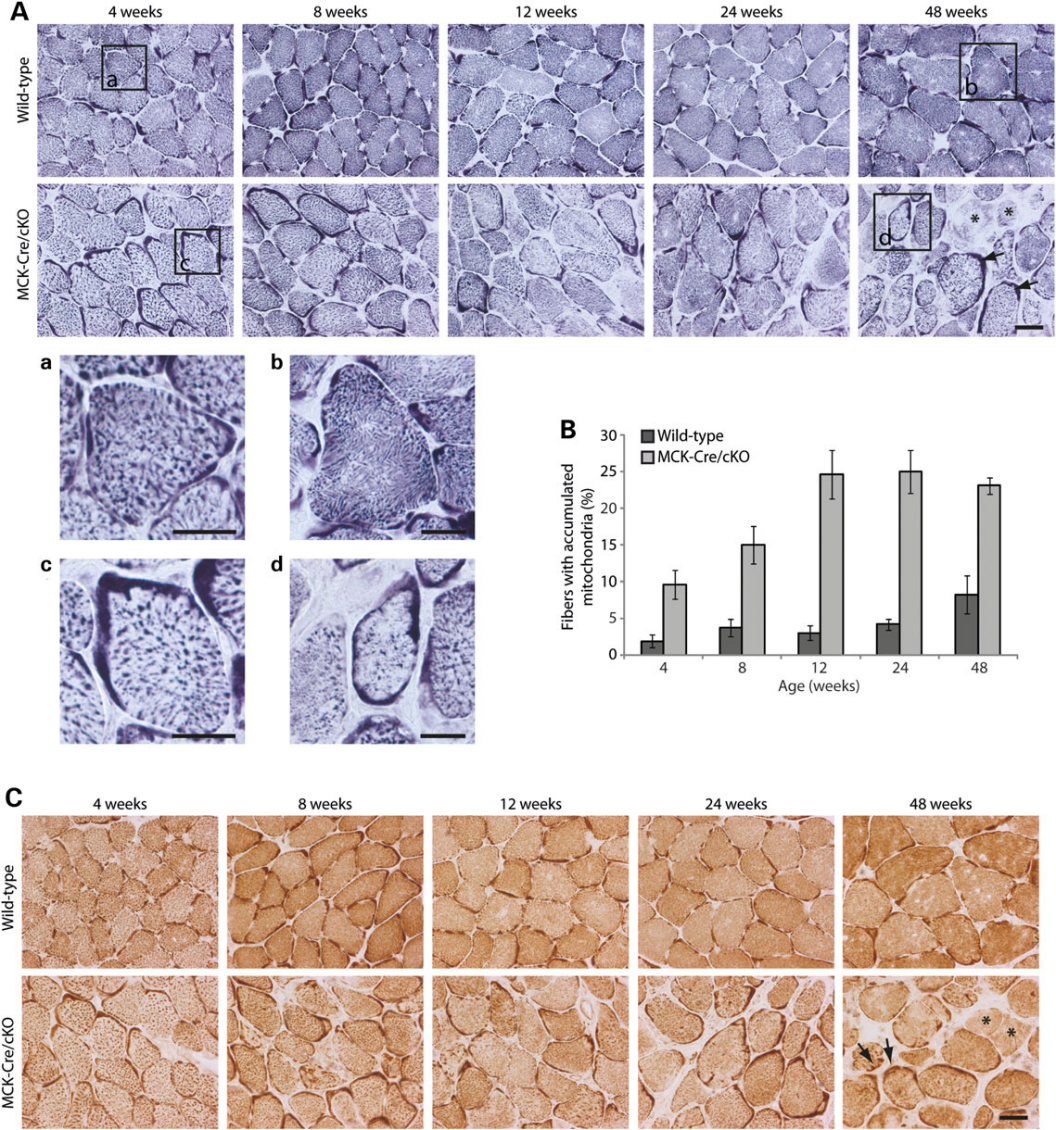
Progression of structural alterations in plectin-deficient skeletal muscle during ageing. (**A**) Soleus cross-sections obtained from 4-, 8-, 12-, 24- or 48-week-old wild-type and plectin-deficient (MCK-Cre/cKO) mice were stained for SDH. (a–d) are magnifications of the boxed areas indicated. Note subsarcolemmal formation of mitochondrial clusters (arrows, lower row, 48 weeks) and fibers with drastically attenuated mitochondrial staining (rubbed-out fibers; asterisks). Scale bars: 50 µm (upper two rows) and 25 µm (a–d). (**B**) Statistical evaluation of muscle fibers displaying submembranous areas with accumulation of mitochondria as shown in (A). Muscle fibers comprising dense SDH-positive areas (extending at least 5 µm into the cytoplasm at their most extended site and spreading over at least 25% of the fiber perimeter) were considered as fibers with accumulated mitochondria. Mean values ± SEM, three experiments. (**C**) Soleus cross-sections obtained from 4-, 8-, 12-, 24- or 48-week-old wild-type and MCK-Cre/cKO mice were stained for COX. Arrows and asterisks, as in (A). Scale bar: 50 µm.

The histochemical staining of muscle cryosections from EBS-MD patients (15,17) and MCK-Cre/cKO mice (14) for succinate dehydrogenase (SDH) or cytochrome oxidase (COX) clearly revealed pathological mitochondrial alterations, but left it open whether these changes represented secondary effects of general muscle degeneration. To assess whether these pathological alterations of mitochondria occurred congenitally or in an age-dependent manner, soleus muscle sections derived from MCK-Cre/cKO and wild-type mice of different age (4, 8, 12, 24 and 48 weeks) were stained for SDH (Fig. 1A). In wild-type fibers of any age, mitochondrial staining occurred mostly granular throughout the cytoplasm, with a slight enhancement of subsarcolemmal regions becoming noticeable in a few sections (Fig. 1A, Boxes a and b). In contrast, in tissue sections from plectin-deficient muscle, mitochondria displayed a less-ordered, more coarse and rather reticular staining pattern (Fig. 1A, Boxes c and d). Moreover, large clumps of aggregated mitochondria became evident in subsarcolemmal regions of many muscle sections; also, the overall intensity of mitochondrial signals in the interior of the fiber appeared reduced (Fig. 1A, Box d). This was in line with ultrastructural analyses showing aggregation of mitochondria in the cytoplasm of MCK-Cre/cKO muscle fibers (14). A statistical analysis of fibers comprising accumulated mitochondria revealed that alterations in mitochondrial morphology were clearly noticeable already in samples from 4-week-old MCK-Cre/cKO mice, becoming more prominent with age, and finally leading to fibers with massive accumulations of mitochondria in subsarcolemmal patches (Fig. 1B). Mitochondrial alterations similar to those illustrated in Figure 1A were observed when corresponding muscle sections were stained for COX (Fig. 1C). Moreover, SDH and COX labeling revealed fibers in which mitochondrial signals were drastically reduced, so-called rubbed-out fibers (Fig. 1A and C, 48 weeks, asterisks). In soleus muscle of 48-week-old MCK-Cre/cKO mice, such fibers accounted for 7% of total fibers (data not shown). These analyses suggested that the mitochondrial alterations observed in MCK-Cre/cKO muscle were an early characteristic, rather than representing a late-onset secondary effect of plectin deficiency, with the severity of the observed phenotype drastically increasing with age.

### Reduced mitochondrial content in plectin-deficient muscle

**Figure 2.**
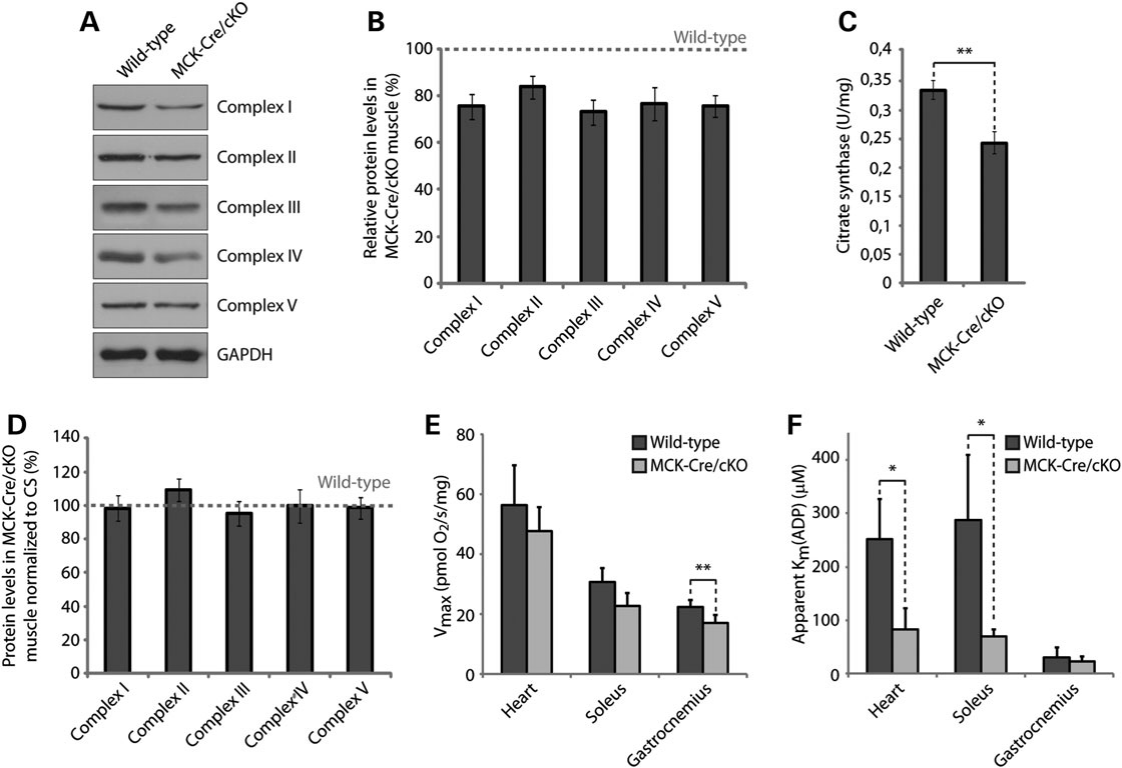
Reduced expression levels of mitochondrial proteins and impaired respiratory function of MCK-Cre/cKO muscle. (**A**) Equal amounts of wild-type and plectin-deficient gastrocnemius muscle lysates were subjected to immunoblotting using antibodies as indicated. GAPDH was used as loading control. (**B**) Signal intensities of immunoblots as shown in (A) were densitometrically measured and normalized to total protein content as analyzed by the Coomassie staining (not shown). Mean values ± SEM, three experiments. Note that the reduced protein levels observed in MCK-Cre/cKO lysates were statistically significant for all mitochondrial respiratory complex proteins assessed (Complexes II and IV, *P* < 0.05; Complexes I, III and V, *P* < 0.01). (**C**) CS activity was measured in wild-type and MCK-Cre/cKO gastrocnemius muscle lysates prepared from 12-week-old mice. Mean values ± SEM, four experiments. (**D**) Relative protein levels as assessed in (B) were normalized to respective CS activity levels as determined in (C). Note that overall protein levels of respiratory complex subunit proteins per mitochondrion remained unchanged in plectin-deficient muscle. (**E**) Respiratory capacities of mitochondria (per milligram wet weight) in permeabilized muscle fibers isolated from heart, soleus or gastrocnemius muscles from wild-type and MCK-Cre/cKO mice. Mean ± SD, three experiments. (**F**) Apparent *K*_m_ for ADP (*K*_m_ADP, affinity of mitochondrial respiration to ADP) in permeabilized muscle fibers isolated from heart, soleus or gastrocnemius muscles from wild-type and MCK-Cre/cKO mice. Mean ± SD, three experiments.

To assess the expression levels of mitochondrial proteins, gastrocnemius muscle lysates prepared from 12-week-old wild-type and MCK-Cre/cKO mice were analyzed by immunoblotting using antibodies to the respiratory Complexes I–V (Fig. 2A). These analyses revealed that, in mutant mice, the protein levels of Complexes I, II, III, IV and V were significantly reduced to 76, 84, 73, 77 and 76% of wild-type levels, respectively (Fig. 2B). To investigate whether these reductions were due to lower levels of total mitochondria in plectin-deficient muscle, we measured the activity of the mitochondrial matrix enzyme citrate synthase (CS) in muscle lysates. CS, the pace-maker enzyme in the citric acid (Krebs) cycle, is commonly used as quantitative marker for the evaluation of intact mitochondria content, because of its stable activity. Indeed, we found its activity to be reduced to 73% in MCK-Cre/cKO compared with wild-type gastrocnemius muscle (Fig. 2C), consistent with the observed diminished levels of respiratory chain complexes. Thus, when normalized to CS activity levels, the protein levels of Complex I–V subunits per mitochondrion were unchanged in MCK-Cre/cKO compared with wild-type samples (Fig. 2D). The observation that, in muscle tissue, plectin deficiency leads to a reduction in mitochondrial content (with normal respiratory complex levels per mitochondrion) is supported by a previous study showing a reduction in the levels of the mitochondrial marker proteins porin (VDAC) and cytochrome *c* in MCK-Cre/cKO muscle (14).

### *In situ* measurements reveal respiratory deficits in plectin-deficient muscle

To investigate whether functional abnormalities of mitochondria could be detected in plectin-deficient muscle tissue, we determined respiratory parameters of mitochondria measured in saponin-permeabilized muscle fibers *in situ*. In this way, the isolation of mitochondria could be avoided, leaving mitochondria in their natural cytoskeletal environment. We performed this analysis with fibers dissected from heart (ventricle), soleus and gastrocnemius muscles, muscle types that are known to differ with respect to mitochondrial content (18). In fibers with high mitochondrial content and thus high oxidative capac­ity, such as myocardium, the maximum respiratory activity (*V*_max_) was ∼3-fold higher than that in the white fibers of gastrocnemius, which contains relatively low levels of mitochondria. In MCK-Cre/cKO heart and soleus muscles, our measurements revealed a tendency toward decreased mitochondrial respiratory capacity (compared with wild type), without, however, reaching statistical significance (Fig. 2E, and Supplementary Material, Table S1), whereas a significant mitochondrial dysfunction was observed in gastrocnemius muscle. Moreover, the apparent Michaelis constant for adenosine diphosphate (ADP) in mitochondrial respiration [app. *K*_m_(ADP)] showed a significant decrease in the cases of oxidative heart and soleus muscles, reflecting an increased affinity of mitochondria for their main substrate, ADP (Fig. 2F, and Supplementary Material, Table S2).

### Mitochondrial alterations are plectin isoform dependent

**Figure 3.**
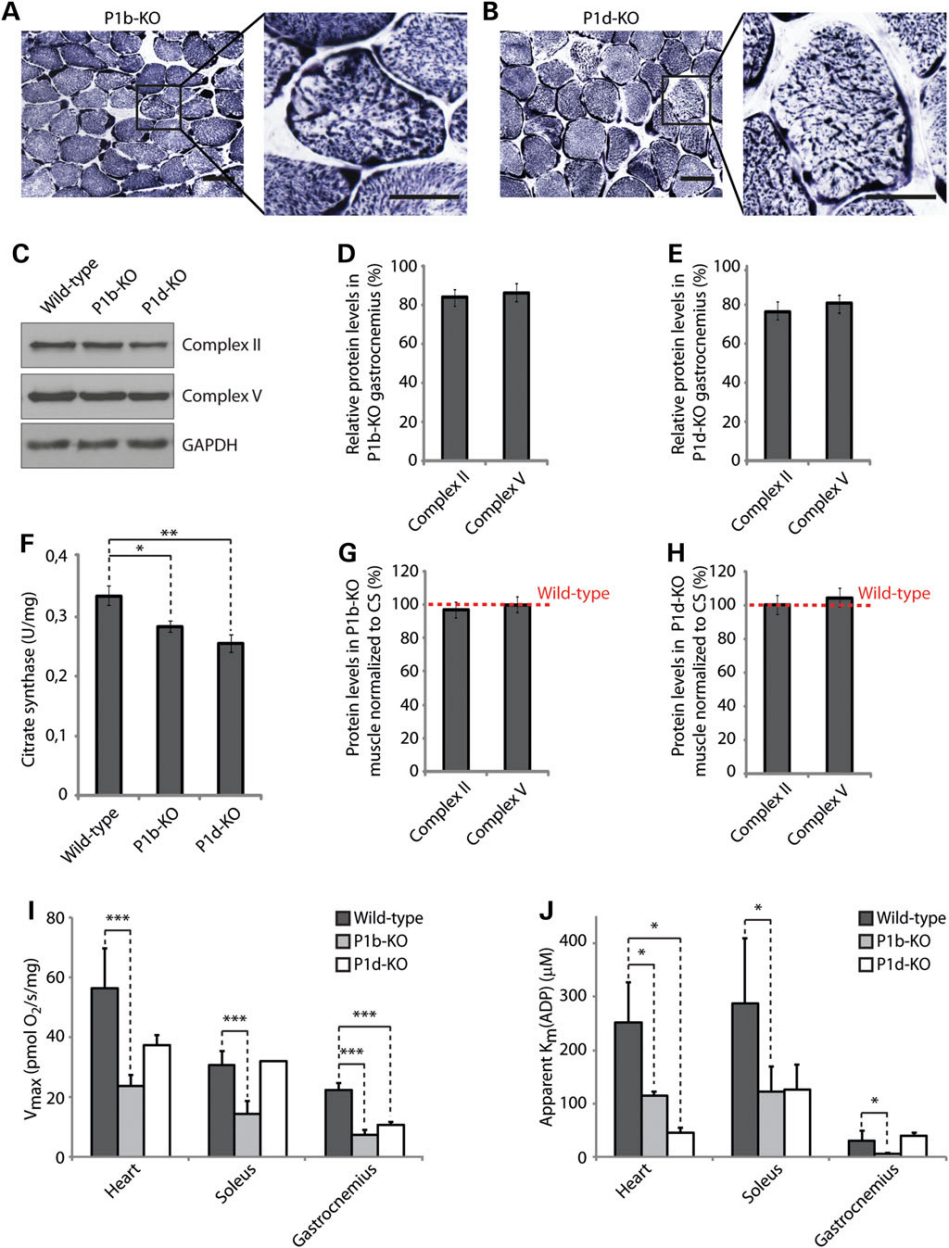
Alterations of mitochondrial functions in plectin isoform-specific knockout tissue. (**A** and **B**) Cross-sections of soleus muscle obtained from 12-week-old isoform-specific P1b (A) and P1d (B) knockout mice were stained for SDH. Boxed areas are shown enlarged. Scale bars: 50 µm (left panels) and 25 µm (right panels). (**C**) Equal amounts of wild-type, P1b-KO and P1d-KO gastrocnemius muscle lysates were subjected to immunoblotting using antibodies as indicated. GAPDH, loading control. (**D** and **E**) Signal intensities of immunoblots as shown in (B) were densitometrically measured and normalized to total protein content as analyzed by the Coomassie staining (not shown). Mean values ± SEM, three experiments. Note that the reduced protein levels observed in P1b-KO and P1d-KO lysates were statistically significant [Complex II, *P* < 0.01 (P1b-KO) and *P* < 0.001 (P1d-KO); Complex IV, *P* < 0.05 (P1b-KO) and *P* < 0.01 (P1d-KO)]. (**F**) CS activity was measured in wild-type, P1b-KO and P1d-KO gastrocnemius muscle lysates prepared from 12-week-old mice. Mean values ± SEM, four experiments. (**G** and **H**) Relative protein levels as assessed in (C) were normalized to respective CS activity levels as determined in (F). Note that the overall protein levels of respiratory complex subunit proteins per mitochondrion remained unchanged in P1b-KO and P1d-KO muscles. (**I**) Respiratory capacities of mitochondria in permeabilized muscle fibers isolated from heart, soleus or gastrocnemius muscles of wild-type, P1b-KO and P1d-KO mice. Mean ± SD, three experiments. (**J**) Apparent *K*_m_ for ADP in permeabilized muscle fibers isolated from heart, soleus and gastrocnemius muscles of mouse lines as indicated. Mean ± SD, three experiments.

Four different isoforms of plectin ensure myofiber integrity by specifically targeting and anchoring desmin IFs to Z-disks (P1d), costameres (P1f), mitochondria (P1b) and the nuclear/SR membrane system (P1) (14). In this study, we focused on P1b, the IF-mitochondrion linker (10) and P1d, which in partnership with desmin cross-links individual myofibrils to each other at the level of Z-disks (14). To investigate whether mitochondrial morphology and functions were specifically dependent on the expression of these isoforms, we analyzed muscle cryosections and tissue lysates derived from isoform-specific knockout mouse lines, lacking either P1b (P1b-KO) (10) or P1d (P1d-KO) (14), while expressing the other isoforms of plectin. SDH-specific staining of cryosections derived from 4-week-old P1b-KO soleus muscle showed mitochondrial patterns that were similar to those of wild-type sections, indicating unaltered distribution and organization of mitochondria at this stage (data not shown). However, in 1-year-old P1b-KO muscle, several fibers displayed aberrant mitochondrial distribution (Fig. 3A). In contrast, cryosections of P1d-KO muscle revealed aggregation and uneven distribution of mitochondria from early on (Fig. 3B, and data not shown). In this case, the staining pattern was reminiscent of that observed in MCK-Cre/cKO muscle, albeit in the latter, mitochondrial aggregates appeared to be larger as shown in Figure 1A and Reference (14). As assessed by staining of ATPase, no alterations in fiber-type distribution were observed for P1b-KO or P1d-KO muscles (Supplementary Material, Fig. S1), indicating that the phenotypes observed were not attributable to fiber-type switches. This was in line with the unaltered fiber-type distribution previously reported for MCK-Cre/cKO muscle (19). When gastrocnemius muscle lysates derived from P1b-KO and P1d-KO mice were analyzed by immunoblotting using antibodies to Complexes II and V (Fig. 3C), a statistical evaluation of protein levels revealed significant reductions of Complex II to 82 and 76% in P1b-KO and P1d-KO tissues, respectively, and of Complex V levels to 85% (P1b-KO) and 80% (P1d-KO), compared with wild-type muscle (Fig. 3D and E). CS activity was reduced to 84% in P1b-KO and to 76% in P1d-KO muscle lysates, compared with wild-type tissue (Fig. 3F). Thus, normalized to the levels of the respective CS activities, the amount of respiratory complexes per intact mitochondrion was found to be unchanged in both P1b- and P1d-deficient muscles (Fig. 3G and H).

Assessing the mitochondrial respiratory capacities of isoform 1b- and 1d-specific knockout tissues, we found that, in both the cases, several muscle types were significantly affected. The most pronounced mitochondrial dysfunctions were found in heart, soleus and gastrocnemius muscles of P1b-KO mice, where respiration was 2- to 3-fold reduced compared with wild type (Fig. 3I). A reduction in respiratory capacity was also found in heart (66% relative to wild type) and gastrocnemius (48%) muscles of P1d-KO mice, whereas no changes were observed in soleus muscles of these mice (Fig. 3I). Taken together, of the different types of striated muscles tested (heart, soleus and gastrocnemius), gastrocnemius showed the most severe mitochondrial dysfunction in all three plectin knockout models (MCK-Cre/cKO, P1b-KO and P1d-KO) analyzed in this study. A comparative survey of all the data on mitochondrial respiratory capacities of wild-type and mutant mouse muscles is shown in Supplementary Material, Table S1.

Similar to the MCK-Cre/cKO mutant mouse line, muscle fibers isolated from heart and soleus muscles of isoform P1d- and P1b-deficient mice showed significantly decreased (2.5- to 5-fold) *K*_m_ values for ADP (Fig. 3J, and Supplementary Material, Table S2). In the case of P1b-KO mice, a significant (4-fold) decrease in *K*_m_ (ADP) values was noticeable also in gastrocnemius, whereas in P1d-deficient mice, this muscle was unaffected. Thus, in general, mitochondrial dysfunctions were more pronounced in P1b deficient than in P1d deficient, or MCK-Cre conditional KO muscle tissues.

### Plectin deficiency leads to spatial rearrangements, shape changes and cytoskeleton decoupling of mitochondrial networks

**Figure 4.**
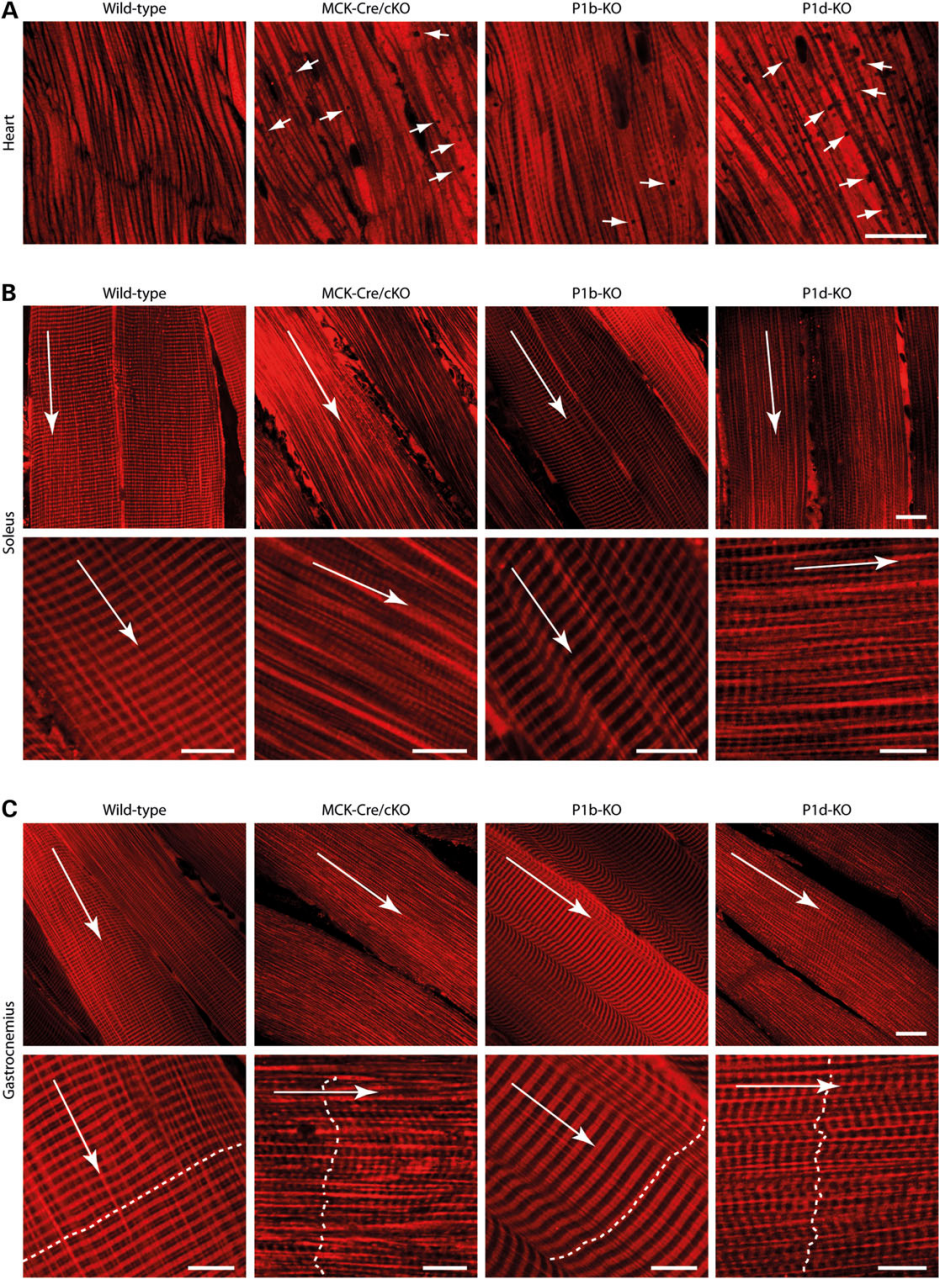
Alterations in mitochondrial morphology in plectin isoform-specific knockout tissues. Confocal images of MitoTracker-stained heart (**A**), soleus (**B**) and gastrocnemius (**C**) muscle tissues derived from wild-type, MCK-Cre/cKO, P1b-KO and P1d-KO mice are shown. In (B) and (C), data are presented as overviews (upper rows) and higher magnifications (lower rows). Long arrows, longitudinal orientation of skeletal muscle fibers. Dashed lines in (C), orientation and position of Z-disks. Note the alterations in mitochondrial positioning and morphology, including ‘black holes’ in the heart (arrows in A), in mutant compared with wild-type fibers. Scale bars: 20 µm (A–C, upper rows) and 10 µm (B and C, lower rows).

*In situ* MitoTracker staining of mitochondria in permeabilized muscle fibers of MCK-Cre/cKO, P1b-KO or P1d-KO mice revealed remarkable differences in mitochondrial network organization compared with wild-type specimens. In the heart, mitochondria-free areas (‘black holes’) became evident in MCK-Cre/cKO as well as in isoform-deficient, particularly, P1d-KO tissues (Fig. 4A). In MCK-Cre/cKO soleus (Fig. 4B) and gastrocnemius (Fig. 4C) muscles, the regular, crystal-like, cross-striated staining pattern of mitochondria characteristic of wild-type fibers (20) was lost and replaced by a much less ordered pattern. In striated muscle of P1b-KO mice, mitochondria appeared to be regularly arranged along Z-disks, but interestingly, the cross striations formed by them showed an increase in width, compared with wild-type fibers. P1d-KO muscle fibers displayed misaligned mitochondrial networks, similar to those of MCK-Cre/cKO fibers, with the distinction of a more striated appearance.

**Figure 5.**
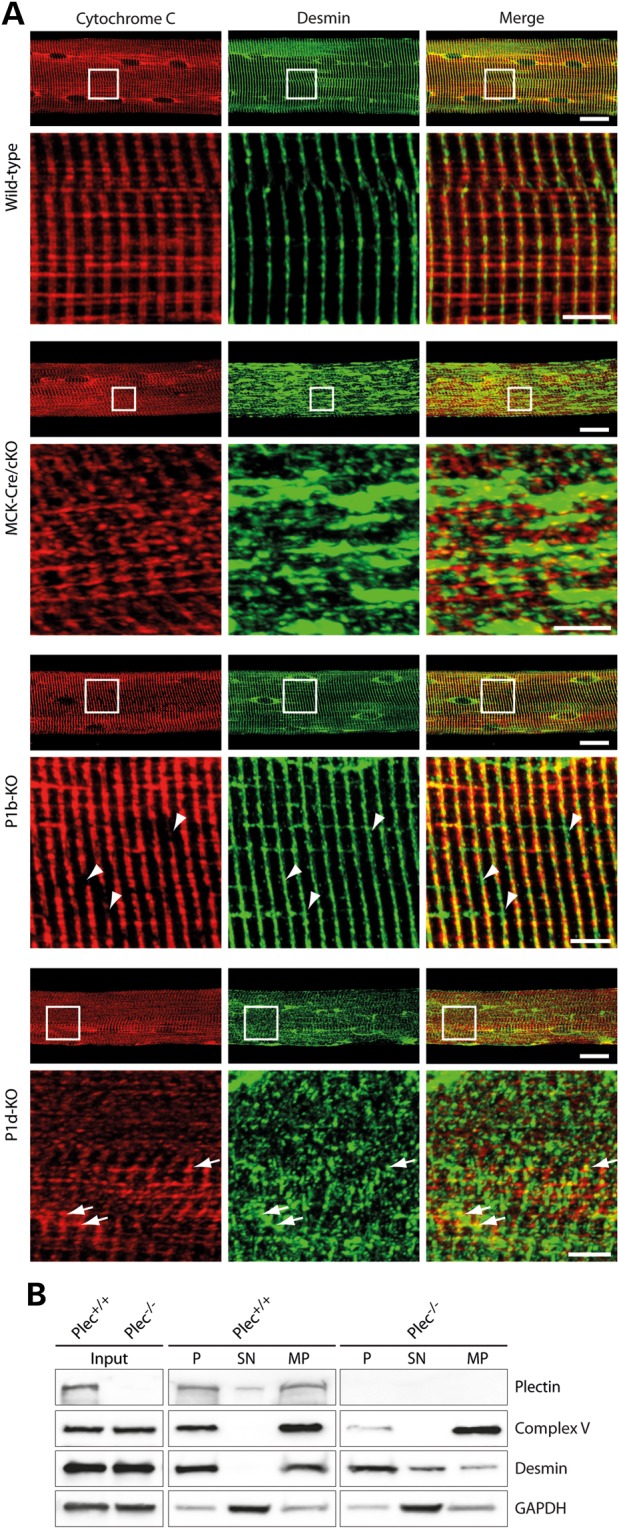
Plectin isoform-dependent alterations in mitochondrial and desmin network architecture. (**A**) Teased EDL muscle fibers derived from wild-type, MCK-Cre/cKO, P1b-KO or P1d-KO mice were co-immunolabeled using antibodies to cytochrome *c* and desmin. Lower panels are magnifications of boxed areas indicated in upper panels. Note collapsed desmin IF networks and randomly oriented mitochondria in MCK-Cre/cKO and P1d-KO fibers. In P1d-KO fibers, contrary to MCK-Cre/cKO fibers, mitochondria were often still in close vicinity to desmin network remnants (arrows). Moreover, note Z-disks regions devoid of mitochondria in P1b-KO fibers (arrowheads). Scale bars: 20 µm (upper panels) and 5 µm (lower panels). (**B**) Immunoblotting of total cell lysates (input) and subcellular fractions derived from *Plec^+/+^* and *Plec^−/−^* myoblasts using antibodies to plectin, Complex V, and desmin. GAPDH was used as loading control. P, pellet (containing nuclei and insoluble cytoskeletal proteins); SN, pooled supernatant fractions of cell homogenates (cytosol) after differential centrifugation; MP, crude mitochondrial pellet. Note that Complex V, the mitochondrial marker protein, could hardly be detected in the insoluble (P) fraction in the absence of plectin, whereas desmin, the cytoskeletal marker protein, was increased in the cytosolic (SN) fraction. One out of three experiments is shown.

To assess the impact of plectin-mediated desmin IF anchorage on mitochondrial network organization, teased extensor digitorum longus (EDL) muscle fibers isolated from wild-type and mutant mice were subjected to double immunofluorescence microscopy for desmin and cytochrome *c*. In control muscle, mitochondria appeared regularly arranged along both sides of (desmin-positive) Z-disks, often extending into the intrasarcomeric space (Fig. 5A). In MCK-Cre/cKO muscle fibers, where the collapsed IF networks generated massive, longitudinally oriented desmin-positive protein aggregates in intermyofibrillar spaces (concurrent with a disintegration of sarcomeric structures), mitochondria no longer appeared to be associated with Z-disks but showed deformation and random orientation. Remarkably, mitochondria did not accumulate in desmin-positive protein aggregates, but seemed to be separated from the IF network remnants. In teased fibers of P1b-KO muscle, the arrangement of mitochondrial networks was similar to that in wild-type fibers, except that their lateral alignment along the Z-disk axes appeared less tight and regular, as suggested by the longer and relatively abundant cytochrome *c*-negative stretches within desmin-positive cross striations (Fig. 5A, arrowheads). In P1d-KO fibers, which, similar to MCK-Cre/cKO fibers, displayed collapsed desmin IF networks in conjunction with smaller and therefore less prominent protein aggregates, mitochondrial networks were unbridledly dispersed, albeit often still in close vicinity to desmin network remnants (Fig. 5A, arrows).

Functionally, plectin is likely to physically link mitochondria to the IF cytoskeleton (10,21), thus contributing to their cellular positioning and mechanical stabilization (14). To confirm this by a biochemical assay, immortalized plectin-null myoblasts (*Plec^−/–^*) and their wild-type counterparts (*Plec^+/+^*) were subjected to subcellular fractionation. Immunoblotting of total cell lysates (Fig. 5B, input) revealed similar expression of the mitochondrial marker protein for Complex V (F1-ATPase α-subunit) and of desmin in *Plec^+/+^* and *Plec^−/−^* myoblasts. Interestingly, upon subcellular fractionation, we found that while in *Plec^+/+^* cells mitochondria were present mostly in the cytoskeletal and the mitochondrial fraction, in *Plec^−/−^* myoblasts Complex V was drastically reduced in the cytoskeletal fraction, indicating that in the absence of plectin mitochondria are less firmly bound to the cytoskeleton. Moreover, in contrast to the *Plec^+/+^* cells, we found desmin to be present in the cytosolic extracts and to be drastically reduced in the mitochondrial fractions of plectin-deficient myoblasts (Fig. 5B). This suggested that, similar to other IF network types, desmin IFs became more soluble in the absence of cross-linking and anchorage through plectin (10,22,23).

**Figure 6.**
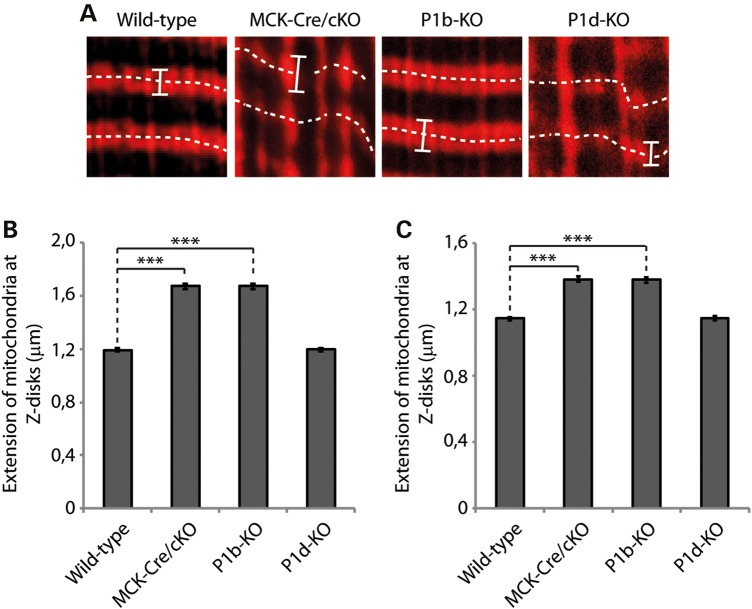
Extension of mitochondrial structures across Z-disk structures in MCK-Cre/cKO and P1b-KO muscles. (**A**) Gastrocnemius muscles were stained using MitoTracker. Bars mark outer rims of mitochondrial structures wrapped around Z-disks. Dashed lines indicate the orientation and position of Z-disks (compare with Fig. 4C). Note the loss of myofibrillar alignment and disorganization of mitochondrial structures in MCK-Cre/cKO and P1d-KO muscles. (**B** and **C**) Statistical analyses of mitochondrial width measurements in gastrocnemius (B) and soleus (C) muscles using ImageJ software. Because of the misalignment of myofibrils in some specimens, particularly MCK-Cre/cKO and P1d-KO muscles, only mitochondria in focus with the optical plane were included in the analysis. Note the increased dimensions of mitochondria in MCK-Cre/cKO and P1b-KO, but not in P1d-KO muscles. Mitochondria from wild-type (*n* = 131), MCK-Cre/cKO (*n* = 129), P1b-KO (*n* = 135) and P1d-KO (*n* = 132) muscle slices were analyzed in three independent experiments.

As MitoTracker-stained muscle tissues and teased fibers immunolabeled for cytochrome *c* both pointed toward an increased size of mitochondria in some of the mutant muscles, we measured the width of the mitochondrial Z-disk wrapping in two types of muscle (Fig. 6). A statistical analysis of the data obtained from gastrocnemius muscle revealed a substantial increase in width for P1b-KO (1.68 µm) and MCK-Cre/cKO (1.67 µm) compared with wild-type (1.19 µm) specimens, equivalent to increases of ∼140% (Fig. 6B); corresponding increases measured in soleus muscles amounted to 121% (MCK-Cre/cKO) and 120% (P1b-KO) (Fig. 6C). As P1d-deficient muscle samples did not show such a trend, these data highlighted a specific role of isoform P1b as regulator of mitochondrial morphology.

### Upregulation of mitofusin-2 in plectin-deficient skeletal muscle

**Figure 7.**
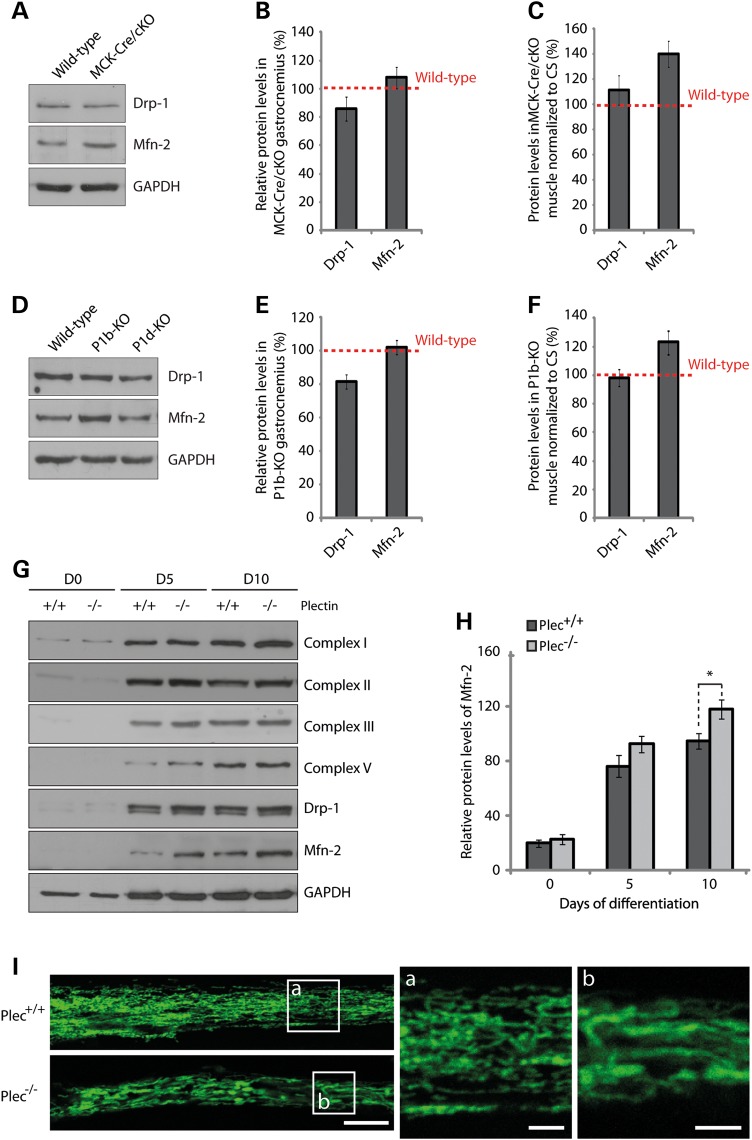
Quantitation of mitochondrial fusion–fission proteins Drp-1 and Mfn-2. (**A**) Equal amounts of wild-type and plectin-deficient gastrocnemius muscle lysates were subjected to immunoblotting using antibodies to Drp-1 and Mfn-2. GAPDH was used as loading control. (**B**) Signal intensities of immunoblots as shown in (A) were densitometrically measured and normalized to total protein content. Mean values ± SEM, three experiments. (**C**) Relative protein levels as assessed in (B) were normalized to respective CS activity levels (Fig. 3C). Note highly increased protein levels of Mfn-2 per mitochondrion in plectin-deficient muscle. (**D**–**F**) as (A–C), except that gastrocnemius muscle lysates from P1b-KO and P1d-KO, instead of MCK-Cre/cKO mice, were subjected to a similar analysis; in (**E** and **F**) only the data for P1b-KO muscles are shown. Note increased Mfn-2 protein levels in P1b-KO but not in P1d-KO muscle lysates. (**G**) Immunoblotting of cell lysates prepared from *Plec^+/+^* or *Plec^−/−^* myoblasts that were either undifferentiated (Day 0) or differentiated for 5 or 10 days. Antibodies used for detection are indicated. GAPDH, loading control. (**H**) Signal intensities of Mfn-2 protein bands as shown in (G) were densitometrically measured and normalized to the total protein content assessed by the Coomassie staining (not shown). Mean ± SEM, three experiments. (**I**) Mitochondrial network organization in differentiated myotubes was visualized by immunofluorescence microscopy of *Plec^+/+^* and *Plec^−/−^* myotubes using antibodies to cytochrome *c*. Note enlarged shape of mitochondria in *Plec^−/−^* compared with *Plec^+/+^* cells. Scale bars: 10 and 2.5 µm (a and b).

To investigate whether plectin deficiency and its ensuing decoupling of mitochondria from the IF network has a direct effect on the mitochondrial fusion/fission machinery, we determined the expression levels of a mitochondrial fission marker, dynamin-related protein-1 (Drp-1), and of the fusion-related protein mitofusin-2 (Mfn-2) in lysates derived from wild-type and MCK-Cre/cKO gastrocnemius muscles (Fig. 7A). The statistical analysis of protein bands revealed a tendency toward reduced levels of Drp-1 (85%) and slightly, but not significantly, increased Mfn-2 protein levels in MCK-Cre/cKO compared with wild-type muscle (Fig. 7B). However, upon normalizing protein levels of Drp-1 and Mfn-2 to mitochondrial content (CS activity; see also Fig. 2C), Mfn-2 protein levels in MCK-Cre/cKO muscle rose to ∼140% of wild-type levels, whereas no significant increase was observed in Drp-1 levels (Fig. 7C). When muscle lysates derived from isoform-deficient mice were analyzed in a similar way, again, an increase in Mfn-2 protein levels (to 121% of wild-type controls) was revealed for P1b-KO lysates (Fig. 7D–F), whereas no changes were noticeable in P1d-KO muscle lysates (data not shown). These observations suggested that, in P1b-KO muscle, mitochondrial fusion processes were increased, similar to MCK-Cre/cKO muscle.

Muscle fibers form by fusion of mono-nucleated single myoblasts into multi-nucleated myotubes. To assess whether cultured myoblasts were mimicking the situation encountered in muscle tissue, and if so, whether their state of differentiation played any role, we measured CS activity and respiratory parameters, including basal cellular respiration, FCCP-stimulated uncoupled respiration and uncoupled control ratio in cell lysates of myotubes. However, neither CS activity nor any of the other measurements revealed significant differences between *Plec^+/+^* and *Plec^−/−^* myotubes (Supplementary Material, Fig. S2A and B). When we assessed by immunoblotting the expression levels of Mfn-2 and Drp-1, along with those of respiratory chain marker proteins (Complexes I–V), in lysates from undifferentiated and differentiated (5 or 10 days) *Plec^+/+^* and *Plec^−/−^* myocytes, we found that the expression levels of respiratory Complex I–V proteins increased upon differentiation in both types of cells, but no differences between *Plec^+/+^* and *Plec^−/−^* cells were noticed (Fig. 7G). Interestingly, however, after 10 days of differentiation, the levels of Mfn-2 were found to be significantly increased in plectin-deficient compared with *Plec^+/+^* myotubes (Fig. 7H). No differences between *Plec^+/+^* and *Plec^−/−^* myoblasts were observed for fission protein Drp-1. Moreover, similar to the situation in undifferentiated single cells (10,11), in *Plec^−/−^* myotubes (differentiated for 10 days), mitochondrial networks appeared enlarged compared with their *Plec^+/+^* counterparts (Fig. 7I). These observations supported the notion that cultured myocytes were indeed mimicking the *in vivo* situation of mitochondria including upregulation of Mfn-2 upon differentiation.

## Discussion

Our study indicates that plectin deficiency in skeletal muscle fibers leads to major alterations of various mitochondrial aspects, including changes in shape and network organization, deficits in respiratory functions and reduced overall content. The structural alterations of mitochondria observed in MCK-Cre/cKO mice occurred already at early stages of development. Thus, it is likely that these alterations were congenital, contributing to the progressive overall phenotype manifestation in plectin-deficient mice and, by implication, EBS-MD patients. Indeed, our study showed that the pathological alterations of mitochondria were significantly increasing with the age of the mice, resulting in multiple fibers displaying reticular patterns of SDH/COX activities, clustering of mitochondria and fibers with attenuated enzyme activities (14,15)—phenotypes found also in patient biopsies (12,15). The probability of the mitochondrial phenotype being congenital was also supported by the data showing that mitochondrial alterations were manifesting not only in adult muscle tissue but also in satellite cell-derived myoblasts and differentiated myotubes.

**Figure 8.**
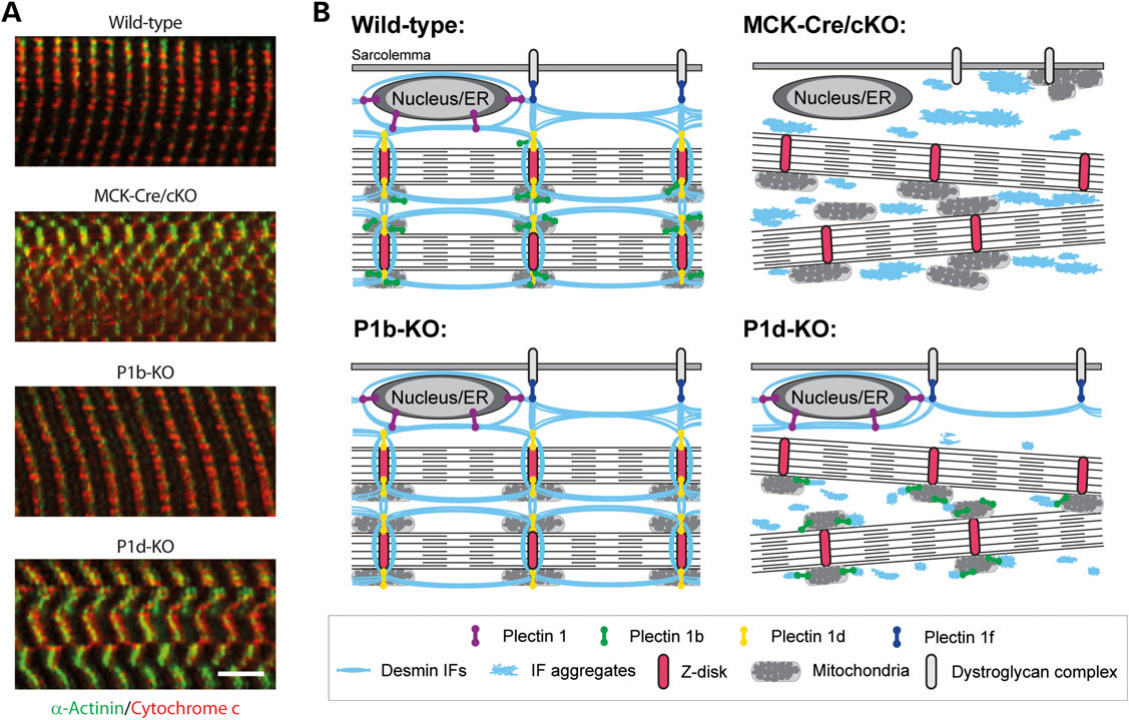
Spatial arrangement of mitochondria in skeletal muscle fibers and working models for phenotypic distinctions between P1b-KO and P1d-KO myofibers. (**A**) Teased EDL muscle fibers derived from wild-type, MCK-Cre/cKO, P1b-KO or P1d-KO mice were co-immunolabeled using antibodies to cytochrome *c* and the Z-disk marker α-actinin. Note that while mitochondria localize along the Z-disks in wild-type fibers, they are dispersed from Z-disks in MCK-Cre/cKO fibers. In P1b-KO fibers, reduced alignment of mitochondria with Z-disks was observed. In P1d-KO fibers, contrary to MCK-Cre/cKO fibers, mitochondria were still associated with the Z-disks. Scale bar: 5 µm. (**B**) Schemes of working models. Phenotypic distinctions are based on this and previous studies (8,9,11,14). In wild-type skeletal muscle, distinct isoforms of plectin bind to desmin IFs and interlink myofibrils with each other at the level of Z-disks (P1d), with the costameric lattice (P1f), with mitochondria (P1b) and with the outer nuclear/endoplasmic reticulum membrane system (P1). In plectin-deficient muscle (MCK-Cre/cKO), desmin IFs detach from Z-disks, costameres, mitochondria and nuclei, thereby causing progressive degenerative alterations including massive desmin aggregation, misalignment of Z-disks, and aggregation and elongation of mitochondria [see also (11,14)]. Loss of mitochondrion-associated P1b leads to elongation of mitochondria, whereas the myofibrillar apparatus remains in order, owing to other plectin isoforms still fulfilling their functions. Deficiency of Z-disk-associated P1d leads to misalignment of Z-disks and generation of small desmin aggregates throughout the sarcoplasm, whereas mitochondria display normal shape and are still associated with myofibrils. Note that the assignment of plectin isoforms to their normal locations in P1b-KO and P1d-KO muscles (schemes in lower row) is predictive but still hypothetical.

The observed structural alterations of mitochondria in plectin-deficient muscle were associated with functional impairments of the organelles, including a significant decrease in the apparent *K*_m_ for ADP in mitochondrial respiration (Fig. 2F, and Supplementary Material, Table S2). This suggests an increase in outer mitochondrial membrane permeability (most probably due to the loss of mitochondria–cytoskeleton interactions associated with impairment of micro-compartmentation effects on several coupled mitochondrial systems), leading to disturbed regulation of normal mitochondrial respiratory function and less effective intracellular energy transfer. The most likely reasons for perturbed membrane permeability could be the disengagement of mitochondria from the desmin cytoskeleton as most clearly observed in MCK-Cre/cKO muscle. The loss of cytoskeleton interactions led also to the partial displacement of mitochondria from Z-disk structures, and the ensuing loss of regular cross-striated mitochondrial staining patterns typical of normal fibers (Fig. 8A, compare upper two images). These results strongly support the emerging view that IFs in general need to be anchored and cross-linked via plectin in order to optimally fulfill their functions (5). Moreover, as mitochondria did not accumulate in desmin-positive protein aggregates and became disconnected from the contractile apparatus (14), as indicated in Figure 8B (upper panels), it seems indisputable that plectin plays a key role in bridging IFs to mitochondrial networks, whereas other mechanisms, including a direct interaction of IF subunit proteins with mitochondria (24), probably play a secondary role. This scenario is in line with the subcellular fractionation of myoblasts, showing that in the absence of plectin mitochondria are more extractable (i.e. more soluble) when unlinked to the IF network (Fig. 5B, upper panels).

Plectin's functional versatility is rooted in the differential cellular targeting of its isoforms, facilitated by their distinct N termini (6,8,25). As we show here for P1d and P1b, the individual ablation of these isoforms in mouse myofibers manifests as distinct phenotypes, including those associated with mitochondria. On the histochemical level, cryosectioned P1d-KO muscle revealed a quite evident pathology, including the misalignment of myofibrils, disorientation of Z-disks, and collapse and aggregation of the desmin IF network system [see also (14)]. In this way, it resembled, but did not mimic, MCK-Cre/cKO skeletal muscle, as its aggregates were smaller and, contrary to the latter case, were found in close vicinity of desmin network remnants and Z-disks (Figs. 5A and 8A). Furthermore, unlike MCK-Cre/cKO fibers, where mitochondria often showed dislocation from Z-disks and aggregation underneath the sarcolemma, in P1d-deficient fibers, most mitochondria were still found in association with Z-disks (Fig. 8A). Thus, it appeared that in P1d-KO fibers, mitochondria-associated P1b was still able to fulfill its function as a linker between the (residual) IF network and mitochondria, as proposed in the hypothetical model shown in Figure 8B (lower panels). On the other hand, in P1b-KO muscle, the structural integrity of myofibers seemed to be maintained, as P1d presumably was still interlinking the contractile apparatus via the desmin IF cytoskeleton (and isoform P1f) to the sarcolemma (Fig. 8B) (9). Nevertheless, a P1b-specific mitochondrial phenotype, manifesting as substantial increase in the dimensions of mitochondria, was evident, the latter being in line with previous observations made with single cells, where we found elongated mitochondrial networks in cultured fibroblasts and myoblasts lacking either all plectin variants (plectin-null) or specifically P1b (10,11).

In addition to affecting mitochondrial morphology, we found that P1b deficiency compromised the respiratory capacity of mitochondria and significantly lowered their *K*_m_ values for ADP *in situ*. Thus, P1b deficiency led to mitochondrial dysfunctions without causing gross disorganization of myofibers, such as misalignment of Z-disks and collapse of the desmin IF network (the hallmarks of P1d deficiency). Furthermore, we propose that uncoupling mitochondria from IFs as a consequence of P1b deficiency, or compromising IF network integrity itself, for example through chemical agents such as polyacrylamide (26), or disruption of IF anchorage at Z-disks (as in the case of P1d deficiency), will lead to mitochondrial dysfunctions. We conclude that mitochondrial disorders observed upon plectin deficiency are, at least in part, isoform specific and therefore must be considered primary rather than secondary effects of plectin mutations. Along these lines, one may anticipate disruptive mutations in plectin's exon 1b or exon 1d (encoding the isoform-specific sequences of P1b or P1d, respectively) to lead to plectinopathies manifesting with mitochondrial dysfunctions alone (P1b), or Z-disk-associated muscular symptoms combined with mitochondrial dysfunction (P1d), similar to site-restricted phenotypic manifestations of recently identified plectinopathies caused by dysfunctional isoforms P1f (27) and P1a (28).

As size, structure and interconnectivity of mitochondrial networks within a cell are determined by the equilibrium of fusion and fission events (29,30), a supposable reason for the elongation of mitochondria in P1b-deficient cells would be an increase in mitochondrial fusion. Although increased mitochondrial fusion rates have not been demonstrated in previous experiments using P1b*^−/−^* and P-null fibroblasts (10), in the present study, we could show that, on the basis of total mitochondrial content, the expression levels of Mfn-2, a key regulator of the mitochondrial fusion process, was increased in both MCK-Cre/cKO and P1b-KO muscles. Because of their simple anatomy, single cells in culture are an attractive model for visualizing mitochondria, but they might not always truly reflect the properties of the organelle in differentiated myotubes and tissue (31). Accordingly, we observed an increase in Mfn-2 protein levels, similar to that seen in MCK-Cre/cKO and P1b-KO skeletal muscle, only in plectin-deficient (differentiated) myotubes, but not in (undifferentiated) myoblasts. Similar to Mfn-2 (32), P1b was shown to be localized in the outer mitochondrial membrane, where it is anchored via its isoform-specific sequence that serves as a single-anchor domain (10). IF-based P1b could either provide a scaffolding platform for proteins that establish and/or maintain mitochondrial morphology on the surface of the organelle (such as Mfn-2), or the interlinkage of mitochondria and IFs mediated by P1b may play a mechanical role in shaping the organelle. Accordingly, IF networks might provide a stable structural backbone required to promote mitochondrial fusion/fission events. Moreover, as plectin was shown to serve as cytoskeletal scaffolding platform for several important signaling proteins in distinct cellular regions (22,33), one might anticipate a possible role of P1b in providing a similar platform at the outer mitochondrial membrane for the recruitment of signaling complexes involved in mitochondrial network dynamics. In this context, it is noteworthy that upon subcellular fractionation, reduced levels of activated PKCδ were found in mitochondrial fractions from P1b*^−/−^*fibroblasts (10). In any case, the lack of a mitochondrion-associated docking site for Mfn-2 in P1b- but not P1d-deficient myofibers could explain why an Mfn-2-specific phenotype was observed only in the case of P1b-KO but not P1d-KO muscle tissues. On the other hand, for those aspects of the mitochondrial (functional) pathology that were shared between P1b-KO and P1d-KO myofibers, the missing direct linkage of mitochondria to an intact IF network might have been a determining factor.

Data supporting these concepts are still preliminary, and future investigations are necessary to elucidate the precise role of mitochondrion-associated P1b. Especially, the molecular mechanisms leading to increased levels of Mfn-2 and presumably to the observed elongation of mitochondrial networks in the absence of P1b remain to be elucidated. Furthermore, the questions of whether there is a direct or indirect association of P1b with Mfn-2, and if, and to what extent such an association may influence human pathology, remain to be investigated.

In conclusion, the present study provides new mechanistic insights into how the cytoskeleton, in particular, the IF network system, affects mitochondrial functions. It is shown that P1b deficiency-related changes in mitochondrial morphology correspond to increased expression levels of Mfn-2. Moreover, we demonstrated that the depletion of distinct plectin isoforms affected the organization, content, function and regulation of mitochondrial networks in different ways. Our study also indicates that the mitochondrial pathology observed in plectin-deficient mouse skeletal muscle, and thus most likely also in plectinopathy patients, is a constitutive mechanism rather than a secondary effect of muscular dystrophy.

## Material and Methods

### Cell culture

Immortalized skeletal myoblasts were derived from *Plec^−/−^* and wild-type littermates, both crossed into a *p53^−/−^* background, and cultivated in Ham's F10 medium, supplemented with 20% fetal calf serum, 2.5 ng/ml basic fibroblast growth factor (bFGF, Promega) and antibiotics, on collagen-coated culture dishes, as described in Reference (11). To induce differentiation, cultures were switched to Dulbecco's modified Eagle's medium containing 5% horse serum.

### Animals

All experiments involving animals were performed according to Austrian Federal Government laws and regulations. MCK-Cre/cKO, P1d-KO and P1b-KO mice have been published in References (10,14). All mouse lines were back-crossed for >10 generations to the C57BL/6 background. Mice bearing floxed plectin alleles (*Plec^f/f^*) in the C57BL/6 background were used as control animals and are referred to as wild type (14).

### Antibodies

For immunoblotting and immunofluorescence microscopy, the following primary antibodies were used: mouse monoclonal antibodies (mAbs) to Complex I (α subcomplex 9, Molecular Probes A31856), mouse mAbs to Complex II (Fp subunit, Molecular Probes A11142), mouse mAbs to Complex III (core II, Molecular Probes A11143), mouse mAbs to Complex IV (subunit I, Invitrogen, clone 1D6E1A8), mouse mAbs to Complex V (α subunit, Molecular Probes A6403), rabbit antiserum (AS) to GAPDH (Sigma, G9545), mouse mAbs to cytochrome *c* (BD Pharmingen, clone 6H2.B4), rabbit AS to desmin (Cell Signaling, 5332), rabbit AS to plectin 46 (25), mouse mAbs to Drp-1 (BD Pharmingen, 611112), rabbit AS to mitofusin-2 (Sigma, M6444) and rabbit AS to α-actinin (Abcam, ab72592). For immunoblot analyses, primary antibodies were used in combination with HRP-conjugated secondary antibodies (Jackson ImmunoResearch Laboratories). For immunofluorescence microscopy, goat anti-rabbit IgG Alexa Fluor 488, goat anti-mouse IgG Alexa Fluor 488, donkey anti-rabbit IgG Rhodamine red and donkey anti-mouse IgG Rhodamine red were used as secondary antibodies (all from Jackson ImmunoResearch Laboratories).

### Immunofluorescence microscopy

For immunofluorescence microscopy of myoblasts, cells were fixed in 4% paraformaldehyde, permeabilized with 0.1% Triton X-100 and immunostained as described in Reference (10). Teased muscle fibers were prepared and processed as described previously (9). Thin sections (5 µm) were obtained from soleus muscle tissue that was frozen in isopentane cooled with liquid nitrogen. The staining procedure (for teased muscle fibers and thin sections) was performed using the MOM Basic Kit (Vector Laboratories) according to the manufacturer's instructions as described in Reference (11). Microscopy was performed using a LSM710 confocal laser scanning microscope (Zeiss) equipped with a Plan-Apochromat 63x 1.4NA objective lens. Images were obtained using the LSM710 module and the Zeiss ZEN software and processed using the Photoshop CS5 (Adobe) software package.

### Preparation of cell and tissue lysates

Cell and tissue lysates for western blot analyses were prepared as described in Reference (11).

### Subcellular fractionation

Myoblasts were harvested in 0.25 m sucrose, 1 mm ethylene glycol tetraacetic acid (EGTA), 10 mm [4-(2-hydroxyethyl)-1-piperazineethanesulfonic acid (HEPES)]–NaOH (pH 7.4) and 0.5% bovine serum albumin (BSA), subsequently homogenized using a Dounce tissue grinder (Wheaton) and centrifuged at 1500*g* for 10 min to sediment nuclei and insoluble cytoskeletal material (P). The supernatant was collected and centrifuged twice at 10 000*g* for 10 min, resulting in a crude mitochondrial pellet (MP) and pooled supernatants (SN).

### Histochemistry of frozen tissue sections

Soleus muscle was snap-frozen in isopentane cooled with liquid nitrogen. SDH and COX stainings of frozen sections (5 µm) were performed as described in Reference (14). Fiber types were assigned based on ATPase staining (pH 4.2, 4.6 and 9.4) following standard protocols. Samples were analyzed using an Axiophot microscope (Zeiss) equipped with a Plan-Neofluar 20x 0.5 NA objective lens. Images were recorded using AxioVision 4.4 software (Zeiss) and processed using Photoshop CS5 (Adobe) software package.

### Measurement of CS activity

Dissected muscles were snap frozen in isopentane cooled with liquid nitrogen, ground in a mortar and homogenized in 0.1 m Tris–HCl buffer, pH 7.0 using a Dounce tissue grinder (Wheaton). Differentiated myotubes were scraped off in 0.1 m Tris–HCl buffer, pH 7.0 and homogenized using a Dounce tissue grinder (Wheaton). CS activities of cell or tissue lysates were measured following the reduction of DTNB [5,5′-dithio-bis (2-nitrobenzoic acid)] by CoA-SH, liberated by the CS reaction in the presence of oxaloacetate and acetyl-CoA according to the protocol published in Reference (34).

### Cellular respiration

Oxygen consumption of the cells was measured with a titration-injection respirometer (Oroboros Oxygraph) in growth medium, before and after the addition of the mitochondrial uncoupler FCCP (4 µm) at 37°C, assuming an O_2_ solubility of 10.5 µmol/L/kPa (1.4 µmol/L/mmHg). DatLab software (Oroboros Oxygraph) was used for data acquisition and analysis. Respiration rates were expressed in pmol of O_2_ per second, per 10^6^ cells. Both endogenous and uncoupled respiration rates were linearly dependent on the cell density in the range of 0.1–6.0 × 10^6^ cells/ml. Also, mitochondria-specific inhibitors blocked respiration confirming that oxygen consumption was due to the mitochondrial respiratory chain (data not shown).

### Analysis of mitochondrial function in permeabilized muscle fibers

Myocardial (left ventricle) or skeletal muscles were dissected according to Reference (35). Mitochondrial respiratory function was measured in saponin (50 µg/ml)-permeabilized muscle fibers by high-resolution respirometry at 30°C, using two-channel titration-injection respirometers (Oroboros Oxygraph) and expressed in pmols oxygen per s, per milligram wet weight. The respiration medium consisted of 110 mm sucrose, 60 mm K-lactobionate, 0.5 mm EGTA, 1 g/l BSA (essentially fatty acid free), 3 mm MgCl_2_, 20 mm taurine, 10 mm KH_2_PO_4_ and 20 mm HEPES, pH 7.1. DatLab software (Oroboros Instruments) was used for data acquisition and analysis. Respiration was stimulated by 1 mm ADP (maximum rate, State 3 respiration) and measured with 10 mm glutamate and 5 mm malate (substrates for Complex I of the mitochondrial respiratory chain). Apparent *K*_m_ for ADP was measured by ADP titration (step-wise increase in ADP concentration) in the range of 0–2 mm ADP. *K*_m_ calculation was performed by hyperbolic fit using ‘Sigma Plot’ software.

### Fluorescent live imaging of mitochondria

In order to analyze the mitochondrial distribution and inner-membrane potential, dissected muscle fibers were incubated for 30 min at room temperature with 0.2 mm MitoTracker Red CMXRos (Molecular Probes). The digital images of MitoTracker fluorescence were acquired with an LSM510 confocal laser scanning microscope (Zeiss) equipped with a 63x 1.4 NA water immersion objective. Images were obtained using the Zeiss LSM Image Browser and processed using the Photoshop CS5 (Adobe) software package. The length of mitochondrial bundles was measured using the ‘Overlay’ and ‘Measure’ options of the Zeiss LSM Image Browser.

### Statistics

Unpaired Student's *t*-test was used; data are given as mean ± SEM; *P*-values *<0.05, **<0.01 and ***<0.001, if not indicated otherwise.

## Supplementary Material

Supplementary material is available at *HMG* online.

*Conflict of Interest statement.* None declared.

## Funding

This work was supported by Austrian Science Research Fund (FWF) Grants P23729-B11, P22080-B20 and I413-B09 (part of Multilocation DFG-Research Unit 1228). Funding to pay the Open Access publication charges for this article was provided by Austrian Science Fund (FWF).

## Supplementary Material

Supplementary Data
