# Supplementary material for: Plectin isoform P1b and P1d deficiencies differentially affect mitochondrial morphology and function in skeletal muscle
Source: Hum Mol Genet. 2015 May 27;24(16):4530–44. doi: 10.1093/hmg/ddv184 (PMC4512624; doi:10.1093/hmg/ddv184)
Supplement: Supplementary Data [file supp_24_16_4530__index.html]

Plectin isoform P1b and P1d deficiencies differentially affect mitochondrial morphology and function in skeletal muscle — Plectin isoform P1b and P1d deficiencies differentially affect mitochondrial morphology and function in skeletal muscle — Supplementary Data 

# Plectin isoform P1b and P1d deficiencies differentially affect mitochondrial morphology and function in skeletal muscle

## Supplementary Data

Supplementary Data

- Supplementary Data - Docx file
