# Supplementary material for: Plectin isoform P1b and P1d deficiencies differentially affect mitochondrial morphology and function in skeletal muscle
Source: Hum Mol Genet. 2015 May 27;24(16):4530–44. doi: 10.1093/hmg/ddv184 (PMC4512624; doi:10.1093/hmg/ddv184)
Supplement: Supplementary Data [file supp_ddv184_ddv184supp.docx]

**Supplementary Material**

**
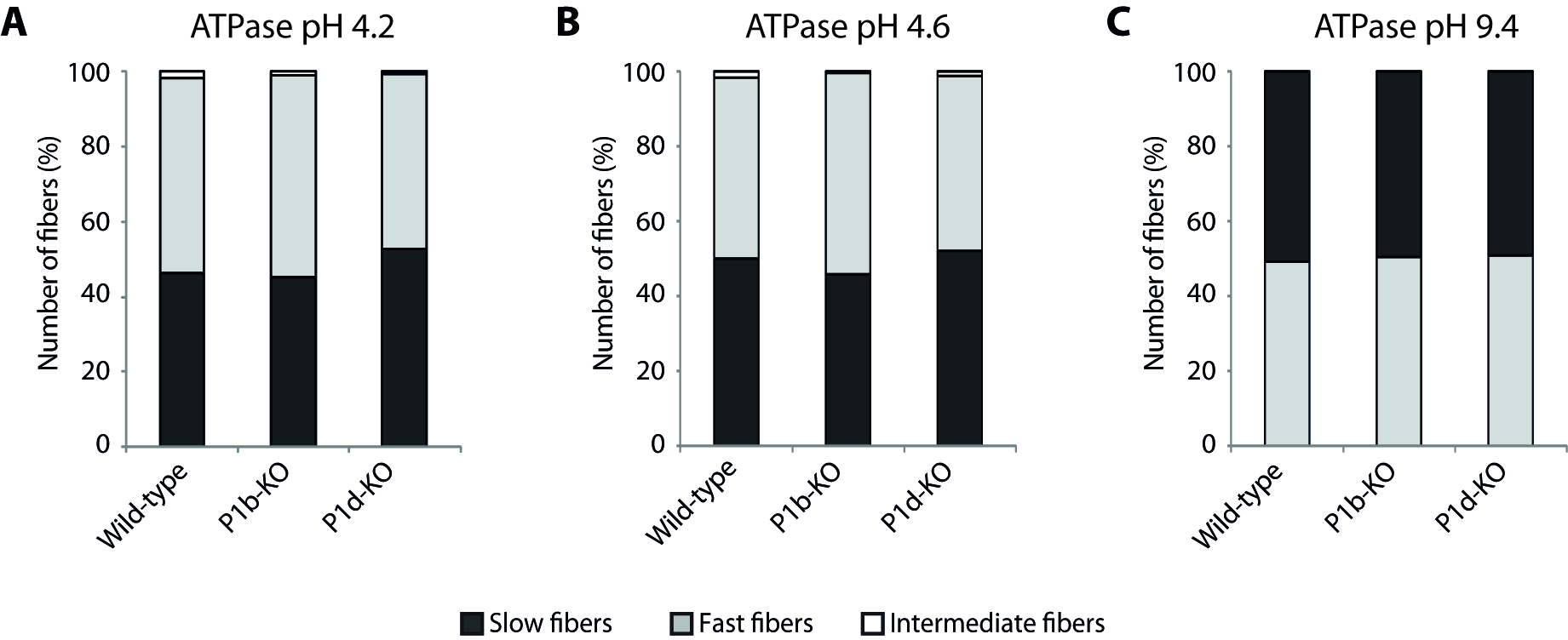
**

**Figure S1. Fiber type distribution in wild-type, P1b-KO, and P1d-KO skeletal muscle.** Fiber type distribution in soleus muscle was determined by staining for ATPase at pH 4.2 (A), pH 4.6 (B), and pH 9.4 (C). Results are shown in percent of total fibers per genotype. Mean values ± SD, n ≥ 11.

**
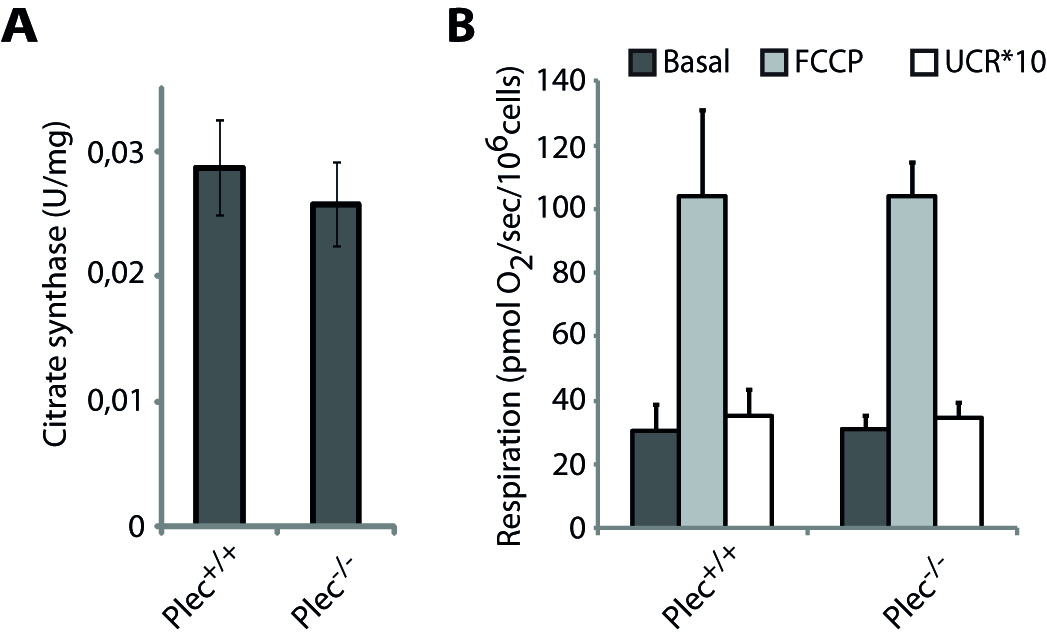
**

**Figure S2. Mitochondrial respiratory function in differentiated myotubes.** (A) Citrate synthase (CS) activity was comparatively measured in differentiated *Plec^+/+^* and *Plec^‑/‑^* myotubes. Mean values ± SEM, 3 experiments. (B) Analyses of respiratory capacities of *Plec^+/+^* and *Plec^‑/‑^* myotubes. Note, no differences were observed in cellular respiration, FCCP-stimulated respiration, or uncoupled control ratio (UCR). Mean values ± SD, 3 experiments.

| **V_max_** | | | | |
| --- | --- | --- | --- | --- |
|  | **Wild-type** | **MCK-Cre/cKO** | **P1b-KO** | **P1d-KO** |
| **Heart** | 56.44 ± 13.36  n=5 | 47.75 ± 8.02  n=4  (*NS*) | 23.83 ± 3.58  n=4  (*P<.,001*) | 37.3 ± 3.4  n=3  (*NS*) |
| **Soleus** | 30.6 ± 4.9  n=5 | 22.7 ± 4.45  n=3  (*NS*) | 14.48 ± 4.25  n=4  (*P<0.001*) | 31.91 ± 0.06  n=3  (*NS*) |
| **Gastrocnemius** | 22.42 ± 2.36  n=8 | 16.98 ± 2.68  n=4  (*P<0.01*) | 7.25 ± 1.67  n=4  (*P<0.001*) | 10.78 ± 0.89  n=3  (*P<0.001*) |

**Table S1.** Respiratory capacities of mitochondria in permeabilized muscle fibers isolated from the heart, m. soleus and m. gastrocnemius of wild-type and various plectin knock­out mice (MCK-Cre/cKO, P1b-KO, P1d-KO). V_max_ is expressed in pmols O_2_ per sec, per mg wet weight. Data are shown as mean ± SD; *NS*, not significant.

| **K_m_(ADP)** | | | | |
| --- | --- | --- | --- | --- |
|  | **Wild-type** | **MCK-Cre/cKO** | **P1b-KO** | **P1d-KO** |
| **Heart** | 251.5 ± 75.7  n=5 | 82.2 ± 39.8  n=4  (P<0.05) | 114.6 ± 7.7  n=4  (*P<0.05*) | 45.7 ± 9.1  n=3  (*P<0.05*) |
| **Soleus** | 287.5 ± 122  n=5 | 69.7 ± 12.5  n=3  (*P<0.05*) | 121.4 ± 48.5  n=4  (*P<0.05*) | 125.2 ± 46.7  n=3  (*NS*) |
| **Gastrocnemius** | 29.8 ± 18.3  n=8 | 22.4 ± 10.3  n=4  (*NS*) | 6.5 ± 0.9  n=4  (*P<0.05*) | 39.6 ± 5.1  n=3  (*NS*) |

**Table S2.** Apparent K_m_ for ADP (K_m_ADP, affinity of mitochondrial respiration to ADP) in permeabilized muscle fibers isolated from the heart, soleus and gastrocnemius of wild-type and various plectin knockout mice (MCK-Cre/cKO, P1b-KO, P1d-KO). Apparent K_m_ for ADP is expressed in µM. Data are shown as mean ± SD. *NS*, not significant.
